# Emerging toolset of three-dimensional pulmonary cell culture models for simulating lung pathophysiology towards mechanistic elucidation and therapeutic treatment of SARS-COV-2 infection

**DOI:** 10.3389/fphar.2022.1033043

**Published:** 2022-12-12

**Authors:** Kai Ni, Bo Che, Chongxin Yang, Youyuan Qin, Rong Gu, Chunhong Wang, Mingzhi Luo, Linhong Deng

**Affiliations:** Changzhou Key Laboratory of Respiratory Medical Engineering, Institute of Biomedical Engineering and Health Sciences, School of Medical and Health Engineering, Changzhou University, Changzhou, Jiangsu, China

**Keywords:** COVID-19, lung organoid, lung-on-a-chip, 3D bioprinting, drug discovery

## Abstract

The ongoing COVID-19 pandemic caused by severe acute respiratory syndrome coronavirus-2 (SARS-CoV-2) poses a never before seen challenge to human health and the world economy. However, it is difficult to widely use conventional animal and cell culture models in understanding the underlying pathological mechanisms of COVID-19, which in turn hinders the development of relevant therapeutic treatments, including drugs. To overcome this challenge, various three-dimensional (3D) pulmonary cell culture models such as organoids are emerging as an innovative toolset for simulating the pathophysiology occurring in the respiratory system, including bronchial airways, alveoli, capillary network, and pulmonary interstitium, which provide a robust and powerful platform for studying the process and underlying mechanisms of SARS-CoV-2 infection among the potential primary targets in the lung. This review introduces the key features of some of these recently developed tools, including organoid, lung-on-a-chip, and 3D bioprinting, which can recapitulate different structural compartments of the lung and lung function, in particular, accurately resembling the human-relevant pathophysiology of SARS-CoV-2 infection *in vivo*. In addition, the recent progress in developing organoids for alveolar and airway disease modeling and their applications for discovering drugs against SARS-CoV-2 infection are highlighted. These innovative 3D cell culture models together may hold the promise to fully understand the pathogenesis and eventually eradicate the pandemic of COVID-19.

## 1 Introduction

The world has been facing coronavirus 2019 (COVID-19) pandemic for 3 years, caused by severe acute respiratory syndrome coronavirus 2 (SARS-CoV-2) (Wang C. et al., 2020; Castiello et al., 2022; Luo et al., 2022). Although most COVID-19 patients present mild or moderate symptoms including cough, fever, fatigue, shortness of breath, and pharyngodynia, critically ill patients of COVID-19 always present severe respiratory complications such as pulmonary edema, acute respiratory distress syndrome (ARDS), and even death (Chen et al., 2020; Guan et al., 2020; Huang et al., 2020). Therefore, COVID-19 which has led to such a global health crisis with huge economic and social impact requires urgent and thorough investigations to elucidate its mechanisms of pathogenesis and explore corresponding therapeutic treatments as well as potential post-disease implications.

However, these investigations so far have been limited by the scarcity of suitable models to mimic the pathophysiological processes of SARS-CoV-2 infection that occur during COVID-19 *in vivo*. Conventional models used to experimentally investigate pathophysiology of human diseases include animal-based models (de Oliveira et al., 2021; Lee and Lowen, 2021). Several kinds of animals including mice (Dinnon et al., 2020), hamsters (Tostanoski et al., 2020), ferrets (Cox et al., 2021), and non-human primates (NHPs) (Corbett et al., 2020; Maisonnasse et al., 2020) have been used to study pathogenesis and host responses associated with COVID-19. It is however difficult to use animal models to recapitulate human physiology and at the same time decipher fundamental molecular mechanisms of host-pathogen interactions, viral replication kinetics, and virus tropism. These animal models are also not very suitable for early-stage drug screening since they are time-consuming and often fail to translate to human trials due to the species difference (Knight, 2008; Konar et al., 2016).

Alternatively, two-dimensional (2D) cell culture systems can be used as experimental models in the study of COVID-19, which are both relatively inexpensive and highly efficient (Duval et al., 2017). But increasing evidence suggests that 2D cell culture systems have inherent drawbacks. For example, 2D cell culture cannot correctly mimic the organ’s *in vivo* architecture and microenvironments (Elsdale and Bard, 1972; Korff and Augustin, 1999; Ghajar et al., 2008). More specifically, the lung is a complex organ including 23 generations of branching airways, multiple cell types, specified tubular three-dimensional (3D) geometry, and cyclic stretch stimulation, which limit the simulating effect using 2D cell culture (Weibel and Gomez, 1962). A recent report has shown that SARS-CoV-2 infection arises from the proximal airways (containing basal, secretory, and ciliated cells) and induces associated inflammation in distal alveoli (containing type I and type II cells) (Morrisey and Hogan, 2010; Borczuk et al., 2020; Tay et al., 2020), which are largely infeasible to be recapitulated by the traditional 2D cell culture systems.

Fortunately, many novel 3D cell culture toolsets such as organoid, lung-on-a-chip, and 3D bioprinting have been developed during the past decade, which provide new platforms for exploring complex pathophysiology of lung diseases such as COVID-19 (de Melo et al., 2021). These 3D cell culture models are indeed gaining increasing momentum to be the primary choice of experimental methods due to their physiological relevance and operational flexibility as well as high-throughput adaptability (Bircsak et al., 2021). Cells cultured in these 3D models exhibit features close to the complex lung conditions *in vivo*, mimic cell-cell and cell-matrix interactions, and reproduce the morphology and function of the lung (Baker and Chen, 2012). Thus, it is evident that 3D cell culture models are advantageous in the investigation of COVID-19 pathogenesis and the development of therapeutic agents to combat the pandemic disease.

In this review, we first describe the characteristics of SARS-CoV-2 and immuno-inflammatory responses related to the viral infection and pathogenesis of COVID-19, and then summarize the 3D cell culture models including the traditional air-liquid interface (ALI) culture and spheroid, and the emerging organoid, lung-on-a-chip (containing organoplate), and 3D bioprinting which may be suitable *ex vivo* models to mimic various vital lung functions in a cell culture dish. We also highlight the possibility of building new robust models to recapitulate different structural compartments of the lung and lung function, in particular, accurately resembling the pathophysiology of COVID-19 *in vivo*. This information may hopefully help investigators to select and/or develop suitable 3D cell culture models for the pursuit of mechanistic understanding and therapeutic treatment of COVID-19.

## 2 Characteristics and immuno-inflammatory responses of SARS-CoV-2

SARS-CoV-2 is a virus with a genome of nearly 30 kb, with 11 open reading frames (ORFs) and 27 viral encoding proteins (Lu et al., 2020). Among them, an array of ORFs, i.e., ORF 3, 6, 7a, 7b, 8, and 10 are the accessory proteins, and the main structural viral proteins include spike glycoprotein (S), envelope glycoprotein (E), membrane glycoprotein (M), and nucleocapsid proteins (N) (Zhou P. et al., 2020; Wu et al., 2020). SARS-CoV-2 enters host cells (ciliated, club, alveolar epithelial type 2 (AT2) cells, vascular endothelial cells, and alveolar macrophages) by endocytosis mediated by the interaction of the S proteins with host receptors such as angiotensin-converting enzyme 2 (ACE2). The S proteins on the envelope of SARS-CoV-2 are cleaved into S1 and S2 subunits (Ke et al., 2020). But only S1 consists of the receptor-binding domain (RBD), which directly binds to the peptidase domain (PD) of ACE2 to gain entry into host cells (Yan et al., 2020). Therefore, the S1 protein/receptor interaction is the critical determinant for the virus to infect host cells. According to various mutation sites in the S protein, six main variants of SARS-CoV-2 viruses have been identified including Alpha, Beta, Gamma, Delta, Lambda, and Omicron. Some variants are more transmissible or easier to escape from immunity than others, which leads to increased transmissibility and a higher viral load in the human body. Garrett and others (Garrett et al., 2022) evaluated asymptomatic carriage in a sub-study of the Sisonke vaccine trial and found that 2.6% of the asymptomatic carriage during the Beta and Delta outbreaks rose to 16% during the Omicron period.

Additionally, some host factors can enable and/or facilitate viral entry. For example, transmembrane protease serine 2 (TMPRSS2) is widely expressed in epithelial cells of the respiratory tract and could activate SARS-CoV-2 in Calu-3 cells (Bugge et al., 2009; Bestle et al., 2020). Furin also plays a critical role in the cleavage activation of SARS-CoV-2 spike proteins (Johnson et al., 2021). Neuropilin-1 (NRP1), which regulates pleiotropic biological processes, facilitates SARS-CoV-2 cell entry and infectivity (Cantuti-Castelvetri et al., 2020; Daly et al., 2020). In brief, these host factors provide an essential mechanism for SARS-CoV-2 infectivity and a scientific basis for targeting infected cells to develop antiviral drugs. Therefore, these host factors’ expression levels in 3D cell culture models may be an important indicator for selecting suitable models for SARS-CoV-2 infection study.

Once SARS-CoV-2 infects the host, both innate and adaptive immune systems initiate to counteract the virus infection. The innate immune response provides the first line of defense against SARS-CoV-2 infection in the airways, *via* various mechanisms for rapid sensing and suppressing of the viral infection. For example, the viral infection is detected by endosomal Toll-like receptor 3&7 (TLR3, TLR7), and melanoma differentiation-associated gene 5 (MDA5) of the innate immune cells in the airways (Totura et al., 2015; Zhou et al., 2021). This subsequently triggers the release of a series of pro-inflammatory factors such as tumor necrosis factor-alpha (TNF-α), and interleukin 1&6 (IL-1, IL-6), which together facilitate the early controlling of the viral infection.

On the other hand, the adaptive immune response provides the second line of defense against SARS-CoV-2 infection, which is enabled by a broader and more finely tuned repertoire of recognition mechanisms for viral infection, involving antigen presenting cells (APCs), CD4^+^ T cells, CD8^+^ T cells and B cells (Sette and Crotty, 2021). More specifically, following SARS-CoV-2 infection, the APCs present viral particles to CD8^+^/CD4^+^ T cells *via* interaction of TCR-MHC I or II, respectively. When exposed to antigens, CD8^+^ T cells release cytotoxic granules that are critical for clearance of virus-infected cells, and CD4^+^ T cells polarize towards Th1 and Th2 cells. Then Th1 cells release IFN-γ to eliminate the virus, and Th2 cells activate humoral immunity (such as B cells) to generate antibodies that neutralize SARS-CoV-2 (Toor et al., 2021). Unfortunately, in severe cases of COVID-19 the potential of this mechanism is significantly limited because the number of APCs is largely reduced (Zhou R. et al., 2020; Qin et al., 2021).

It is obvious that a deficiency of the immune responses would give an opportunity for viruses such as SARS-CoV-2 to freely complete their RNA replication process and subsequent release of the genetic materials, and ultimately result in reassembling and release of large amounts viruses (Jiang et al., 2021). However, an excessive immune response may also trigger excessive production of inflammatory cytokines, a phenomenon known as the cytokine storm. For example, in critically ill patients of COVID-19 the expression of inflammatory cytokines including IL-2, IL-6, IL-7, IL-10, IP-10, MCP-1, TNF-α, and IFN-γ has been shown to be excessively elevated, and this kind of cytokine storm is thought as the main cause of multi-organ failure and death in COVID-19 patients (Castelli et al., 2020; Hu et al., 2021; Luo et al., 2022).

## 3 3D cell culture models to mimic lung in study of SARS-CoV-2 infection

**FIGURE 1:**
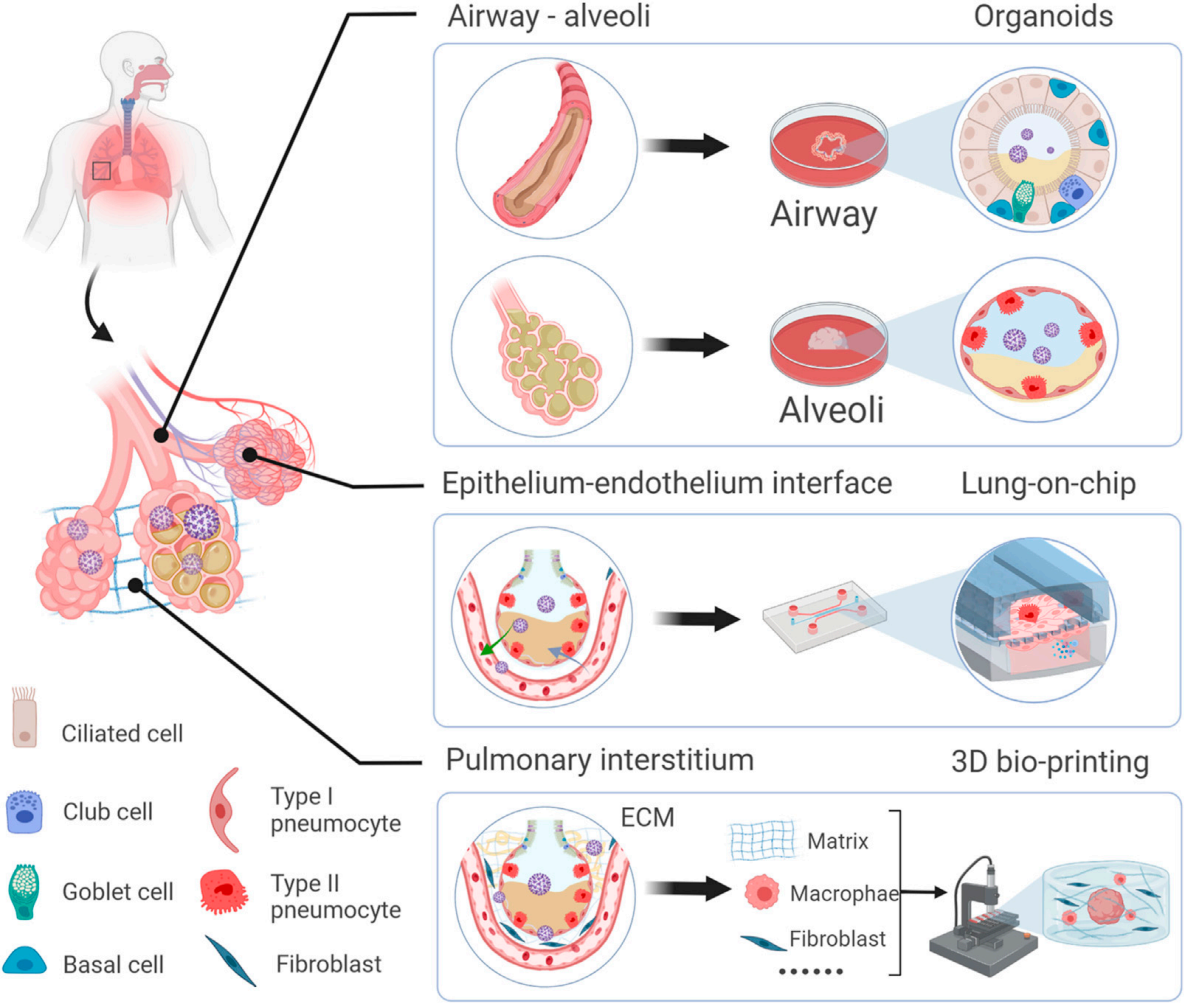
Schematic representation of novel 3D models to mimic *in vivo* the physiology and pathophysiology of different lung regions. Different 3D cell culture models (organoid, organ-on-a-chip, and 3D bioprinting) have their own advantages which could mimic the structure and function of different lung regions (airway, alveoli, a thin epithelial-endothelial barrier, and pulmonary interstitium).

With increasing studies of COVID-19, the importance is acknowledged that different lung regions (airway, alveolus, a thin epithelial-endothelial barrier, and pulmonary interstitium) play different essential roles in the pathogenesis and development of COVID-19. Therefore, 3D cell culture models (ALI culture, spheroid, organoid, organ-on-a-chip including organoplate, and 3D bioprinting) which could mimic different structural compartments of the lung and lung function may be more suitable for studying different physiopathological processes of COVID-19 (Figure 1). In addition, these 3D cell culture models have different advantages in mimicking the physiology and pathology of diseases (Table 1). For example, the ALI approach is primarily used to mimic the epithelial air-liquid interface environment of respiratory tract in the lung. Spheroids are mainly used in study of tumor growth such as in lung cancer. Organoids are self-assembled constructs that can “freely” grow in resemblance to natural development. Organ-on-a-chip has bridged microfluidic technology and living cells, resulting in a dynamic biomimetic device (de Oliveira et al., 2021). 3D bioprinting models can print more complex constructs and mimic complex anatomical structures.

**TABLE 1:** Summary of the different *in vitro* models with advantages and disadvantages.

Culture model	Advantages	Disadvantages
Animals	Long-term effects of drugs	Time consuming
	Whole living organism	Limited high-throughput experiment
	Complex heterotypic microenvironment	Prominent differences between animals and human
2D cultures	Simple	Limited ECM production
	Low-cost	High stiffness of surface
	Commercially available	2D flat culture
ALI cell culture	Co-culture with epithelial and stromal cells	Cell-matrix interactions
	Achieve airway complete epithelium	Dynamic physical factors
Spheroids	Maintain intrinsic phenotypic properties	Diffusion gradient and lack of nutrients in the core
	Organotypic model for cancer study	Uniform size
Organoids	Cell-cell and cell-matrix interactions	Establishment can take long
	Follows developmental stages similar to actual organs	No control on cellular arrangement and growth
	Highly relevant morphology and phenotype	Limited in size
Lung-on-chip	Physiologically relevant air and liquid flows	Commonly used material for chips (PDMS) is hydrophobic and can absorb drugs
	Small volumes required	Limited throughput, especially when combined with flow
	Very controlled environment	Requires special equipment and, depending on design, access to special facilities
3D bioprinting	Accurately arranges the cells	Cell viability varies based on cross-linking and the shear stress of passing through the nozzle
	Enhances cell viability, function, migration and self-assembly	Expensive material of limited availability
	Different printing strategies available	Time-consuming, limited throughput

Abbreviations: ECM, extracellular matrix; PDMS, polydimethylsiloxane.

### 3.1 3D cell culture to mimic ALI of the lung

The respiratory system provides an ALI interface to protect the body from invasion by inhaled pathogens that are commonly encountered in the environments. This interface can not be mimicked by the submerged 2D cell culture models, until the development of 3D ALI culture models (Whitcutt et al., 1988). 3D ALI culture mainly consists of an outer plastic culture dish and an inner porous membrane insert (usually in a 6-well plate format with a pore size of 0.4 μm). Cells are cultured on the insert, and once confluent (often in 2–4 days), the medium is removed from the apical aspect of the insert, forming an ALI. Consequently, this cell culture method has been used to mimic ALI in the lung with different types of airway epithelial cells, including ciliated cells, club cells, goblet cells, and basal cells, to self-assemble into a pseudo-stratified columnar epithelium (Ghio et al., 2013). These functional epithelia provide crucial functions for maintaining airway tissue integrity and homeostasis by secreting inflammatory mediators and antimicrobial peptides (Tam et al., 2011).

By using this traditional 3D cell culture, Mulay et al. (Mulay et al., 2021) discovered that SARS-CoV-2 infected ciliated cells at a significantly higher rate than goblet cells in the proximal airway epithelium. In addition, Mullen and others (Mullen et al., 2021) found in ALI cultures that SARS-CoV-2 infection increased pyruvate carboxylase (PC) and mTORC1 activity and inhibition of mTORC1 could reduce SARS-CoV-2 replication. These studies collectively demonstrate that ALI culture system is an important platform for screening therapeutics targeting airway cells of SARS-CoV-2 and related antivirals for COVID-19.

### 3.2 Spheroids culture to mimic alveoli of the lung

Spheroids allow cell colonies to self-assemble and form aggregates of 3D microtissues (Ryu et al., 2019). The process of spheroid formation is affected by adhesion and differentiation of cells and various factors, including gradients of nutrients, oxygen, and growth factors in cell culture medium.

Spheroids are mainly used for cancer studies because it shows some characteristics similar to tumor microenvironments such as hypoxia and tumoral cell-to-cell interaction (Nigjeh et al., 2018). But it has also been used as 3D cell cultures for recapitulating the anti-viral drug responses in airway cells. For example, Ebisudani et al. (Ebisudani et al., 2021) established and validated a long-term culture of alveolospheres that could be used as an efficient drug testing platform for the development of therapeutic agents to combat viruses such as SARS-CoV-2.

### 3.3 Organoids to mimic different regions of the lung

Novel 3D cell culture systems such as organoids have emerged recently with great potential in biomedical research. Organoids are 3D structures derived from stem/progenitor cells in specific biomaterials that can be differentiated to generate 3D structures containing multiple cell types and assembles that resemble the organization and functions of specific tissue/organs. Therefore, the development of stem cell technology has been central to the formation and progression of organoids. For example, Takahashi and Yamanaka (Takahashi and Yamanaka, 2006) demonstrated the creation of pluripotent cells directly from mouse embryonic or adult fibroblasts by introducing four transcription factors. Soon afterward, Sato et al. (Sato et al., 2009) reported the formation of 3D structures of single-sorted Lgr5^+^ stem cells in Matrigel. Ever since, there have been many organoids successfully generated and used in different fields.

Lung organoids can be established from induced pluripotent stem cells (iPSCs) or airway epithelial progenitor cells such as AT2 cells or basal cells under optimal conditions. The processes of different cell sources to generate lung organoids are described in Table 2. iPSCs-derived organoids contain AT1, AT2, and epithelial cells, and recapitulate the structure and function of the alveolus or airway when exposed to suitable induction signals (Takahashi et al., 2007; Leibel et al., 2020).

**TABLE 2:** The process of different cell source to generate lung organoids.

Cell source	Medium		Treatment period	Matrigel concentration	Results/Application	Refs
iPSCs	DE	CHIR99021 and Activin A in RPMI1640	Day 1	100% matrigel mixed with LPs (Corning, #354234)	hAWOs contain goblet cells (MUC5AC^+^), basal cells (P63^+^), ciliated-like cells (FOXJ1^+^), and proliferating cells (CDK1^+^TOP2A^+^)/Serve as a platform to perform a high content screen for blocking SARS-CoV-2 infection	Duan et al. (2021)
		Activin A and 2% FBS in RPMI1640	Day 2–3			
	AFE	Dorsomorphin dihydrochloride, SB431542, IWP2	Day 4–5			
	LPs	CHIR99021, human BMP4, all-trans retinoic acid	Day 6–14			
	hAWOs	FGF10, FGF2, dexamethasone, 8-bromo-cAMP, IBMX	D15-			
ESCs	DE	CHIR99021 and Activin A in RPMI1640	Day 1–3	100% Matrigel mixed with AFE (BD Biosciences, #356237)	hAWOs contain basal cells (P63^+^), ciliated cells (acetylated TUBULIN, a-TUB^+^), club cells (CC10^+^), and goblet cells (MUC5AC^+^) hALOs contained AT2 cells (SPC^+^) and AT1 cells (PDPN^+^)/Serve as a pathophysiological model to investigate the underlying mechanism of SARS-CoV-2 infection	Pei et al. (2021)
	AFE	Noggin, FGF4, CHIR99021 and SB431542 in Advanced DMEM/F12	Day 3–7			
	VAFE	Human BMP4, all-trans retinoic acid, CHIR and 1% Glutamax in DMEM/F12	Day 8–14			
	LPs	CHIR99021, FGF10, KGF, DAPT	Day 15–21			
	hAWOs	Dexamethasone, 8-Br-cAMP, 3-isobutyl-1-methylxanthine, KGF, B-27 supplement, BSA and 0.1% ITS premix in Ham’s F12	Day 22–28			
	hALOs	CHIR99021, SB431542, Dexamethasone, 8-Br-cAMP, 3-isobutyl-1-methylxanthine, KGF, B-27 supplement, BSA and 0.1% ITS premix in Ham’s F12	Day 22–28			
HBECs derived from airways tissue	hAWOs	R-Spondin 1, FGF 7, FGF 10, Noggin, A83-01, Y-27632, SB202190, B27 supplement, N-Acetylcysteine, Nicotinamide, GlutaMax and HEPES in Advanced DMEM/F12	Cultured for 21 days	50% growth factor reduced Matrigel mixed with HBECs (Corning)	hAWOs contain basal cells (KRT5^+^) and goblet cells (MUC5AC^+^)/Serve as a physiologically relevant airway epithelial model to investigate SARS-CoV-2 therapeutics	Chen et al. (2022)
AT2s derived from primary lung tissue	hALOs	CHIR99021, BIRB796, Y-27632, SB431542, EGF, EGF10, B27 supplement, N-Acetyl-L-cysteine, Heparin, Antibiotic-Antimycotic, GlutaMax and HEPES	Cultured for 10–14 days	50% Matrigel mixed with AT2s (Corning, #354230)	hALOs contained AT2 cells (SFTPC) and AT1 cells (AGER)/Serve as an alveolar epithelium model for understanding human respiratory diseases	Katsura et al. (2020)
Tracheal epithelial cells derived from mouse trachea	AWOs	Insulin, transferrin, cholera toxin, EGF, bovine pituitary extract, 5% FBS and retinoic acid in DMEM/F12	Cultured for 10–14 days	100% growth factor-reduced Matrigel mixed with cells (Corning)	AWOs fused and formed interconnected lumina in a free-floating condition/Serve as ideal modular units for the biofabrication of biomimetic organs	Liu et al. (2021)
HBECs purchased from company	hAWOs	PneumaCult-ALI Maintenance Medium (Stemcell Technologies)	Cultured for 21 days	40% on the bottom, 5% mixed with cells	hAWOs are able to self-organize and mature toward lung tissue-like structures/Serve as a novel tool for studying disease-relevant cellular and molecular function and ectopic transplantation	Tan et al. (2017)

Abbreviations: DE, definitive endoderm; AFE, anterior foregut endoderm; VAFE, ventralized anterior foregut endoderm; LPs, lung progenitors; hAWOs, human airway organoids; hALOs, human alveolar organoids; AT2s, human alveolar epithelial type-2 cells; EGF, epidermal growth factor.

AT2 cells reside in the alveoli which are characterized by the production of pulmonary surfactant proteins and can behave as alveolar stem cells during repair after injury, repopulating both AT1 and AT2 cells (Diem et al., 2020). Basal cells, characterized by the marker of the transcription factor Trp63, the cytokeratin Krt5, and integrin alpha 6, are one type of proximal airway epithelium (accounting for ∼30%), which can differentiate into secretory and ciliated cells (Lambrecht and Hammad, 2012; Barkauskas et al., 2017; Bilodeau et al., 2021). Compared to iPSCs, alveolar or airway organoids based on AT2 cells and basal cells from adults present the physiological dynamic consistent with normal *in vivo* or human patients, especially for COVID-19 (Wang et al., 2021).

**FIGURE 2:**
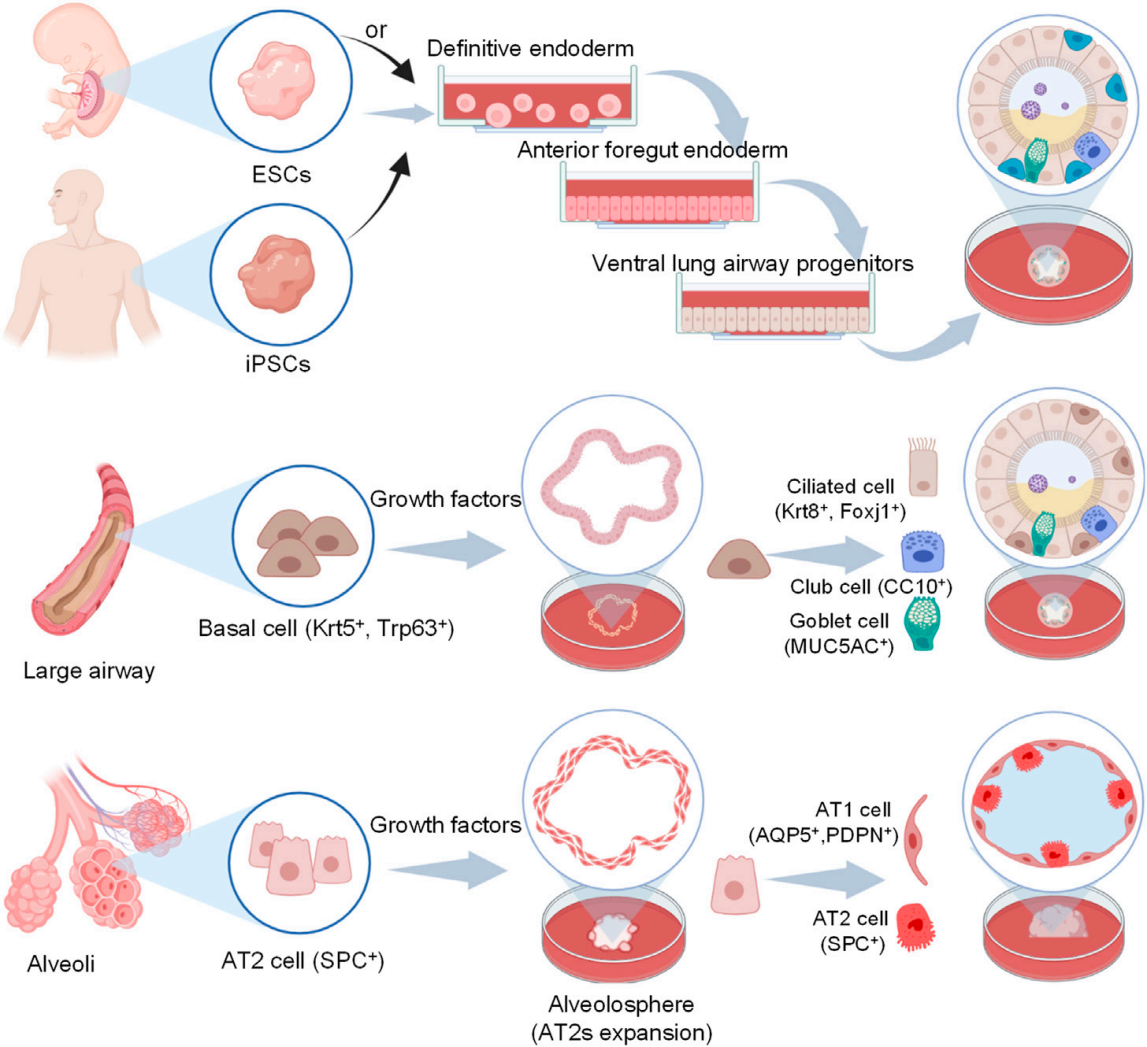
Schematic representation of human airway and alveoli organoids developed from different origin cells and cultivation include particular steps. For iPSCs, they first generate definitive endoderm (DE), then anterior foregut endoderm (AFE) and lung progenitors (LPs) with different additives, finally yield lung organoids *via* 3D culture using Matrigel (Barkauskas et al., 2017). For basal cells and AT2 cells, they maintain under suitable conditions and self-organize in a more natural manner with Matrigel.

Due to the need to mimic the effects in different lung regions, alveolar and airway organoids have been developed to mimic the airway and alveoli, respectively. And a simplified visual description of the main steps to generate lung organoids of different origin cells is in Figure 2.

#### 3.3.1 Alveolar organoids

Though the SARS-CoV-2 virus typically initiates in the proximal airways, severe symptoms of COVID-19 arise from infection and associated inflammation in the distal alveoli, which has strikingly different physiology from that of the proximal airways (Katsura et al., 2020; Mulay et al., 2021). Previous studies have demonstrated that ACE2 is essential for SARS-CoV-2 entry, indicating that AT2 cells with a high expression of ACE2 proteins are significant targets of SARS-CoV-2 infection (Zhao B. et al., 2020). To explore the response of lung cells (particularly AT2 cells) to the SARS-CoV-2 infection, Han and collaborators (Han et al., 2021) developed an alveolar organoid model derived from iPSCs. Similar to what is seen in COVID-19 patients’ lung autopsy tissues (Blanco-Melo et al., 2020), their results revealed that robust induction of chemokines (such as rheumatoid arthritis, TNF signaling, and IL-17 signaling) upon SARS-CoV-2 infection. Also, these AT2-derived organoids were able to differentiate into AT1 cells and could facilitate diverse investigations of pulmonary pathogens, including SARS-CoV-2 infection (Salahudeen et al., 2020).

In addition, the expression of ACE2 could be modulated by the activation of different pathways. When treated with particulate matter (PM2.5), Kim et al. (Kim et al., 2020) proved that ACE2 significantly upregulated in alveolar organoids. On the contrary, androgen signaling inhibition reduced ACE2 expression and protected lung organoids against SARS-CoV-2 infection (Samuel et al., 2020).

#### 3.3.2 Airway organoids

Since the proximal airway regions are the first target of SARS-CoV-2 infection, airway organoids are suitable for exploring the interaction of SARS-CoV-2 with proximal airway cells. To accurately mimic proximal airway physiological conditions during SARS-CoV-2 infection, (Chen et al., 2022) used human airway basal cells to generate airway organoids, in which basal cells differentiate into ciliated cells, goblet cells, and club cells. Furthermore, they compared ALI cultures and airway organoid cultures and acknowledged that the latter expressed high levels of ACE2 and TMPRSS2, which are highly susceptible to SARS-CoV-2 infection and promote inflammatory cytokine responses (Marescotti et al., 2019; Wang J. et al., 2020; Chen et al., 2022).

Furthermore, to observe cellular dynamic changes similar to clinical features in COVID-19 patients, (Xu et al., 2021) developed airway organoids derived from patient biopsy residues. Therefore, these airway organoids can be used to investigate the tissue-specific SARS-CoV-2 infection, host responses, and viral infection inhibitors.

To investigate whether multibasic cleavage site (MBCS) can alter protease usage during entry and which entry pathway is taken by SARS-CoV-2, (Mykytyn et al., 2021) found that SARS-CoV-2 spike MBCS increases infectivity and serine protease usage on human airway organoids based on collagen-coated transwell inserts. In addition, (Kastenhuber et al., 2022) demonstrated that coagulation factors, including factor Xa and thrombin, increase SARS-CoV-2 infection in human lung organoids derived from iPSCs. These data indicate that these hose factors can directly cleave SARS-CoV-2 spike, which is important to promote viral entry into airway epithelia.

Together, these data demonstrate that organoid models could accurately mimic alveolar and airway cellular composition to provide a valuable platform for screening new drugs to identify candidate COVID-19 therapeutics.

#### 3.3.3 Lung organoids

iPSCs-derived lung organoids are indisputably a fast-moving field due to the unique property of unlimited self-renewal capacity (Kolagar et al., 2020; Sharma et al., 2020). To date, the most classical differentiation protocols first generate definitive endoderm (DE), then anterior foregut endoderm (AFE) and lung progenitors (LPs), and finally yield lung organoids using 3D Matrigel. Tiwari et al. (2021) developed human lung organoids derived from iPSCs to investigate viral pathogenesis. iPSCs were differentiated into definitive endoderm, lung progenitor cells, then an epithelial-like structure with surrounding mesenchymal cells (labeled with smooth muscle actin and acetylated tubulin) by day 60, and subsequently a pseudostratified epithelial structure with P63^+^ basal-like cells, FOXJ1^+^ ciliated cells, and structural alveolar type 1 & 2 cells.

In addition, 3D cell culture models such as organoids are suitable for further understanding immuno-inflammatory responses associated with SARS-CoV-2 infection, which is crucial for effective control and clearance of the virus. For example, iPSCs-derived lung organoids have been used to determine the early cellular response to SARS-CoV-2 infection, particularly the change in the expression level of inflammatory factors with 48 h infection (Pei et al., 2021). The results of RNA-sequencing analysis show that several inflammatory factors, including IL-6, TNF, CXCL8, CXCL2, CXCL3, CXCL10, CXCL11, and NF-κB were upregulated, which is consistent with the clinically observed phenomenon in COVID-19 patients (Huang et al., 2020; Wilk et al., 2020).

Therefore, iPSCs-derived lung organoids containing the component and structure of proximal airways and distal alveoli, can be used for revealing cell/tissue-specific SARS-CoV-2 Infection and host responses in the whole lung (Dye et al., 2015).

### 3.4 Lung-on-a-chip models to mimic epithelium-endothelium interface

Although lung organoids are promising tools to elucidate the pathophysiological mechanisms of COVID-19, a significant limitation of them is the absence of vasculature, and could not mimic the interaction of alveoli-capillary networks and related gas exchange in the lung (Barkauskas et al., 2017). The gas exchange process in the human body depends on the direct interaction between a monolayer alveolar epithelia lining the alveoli and a monolayer endothelial cell lining the capillary network, which allows for diffusive gas exchange and prevents plasma fluid entry into the alveoli (Weibel, 2017; Bernard et al., 2020). With growing interest in COVID-19, the importance of crosstalk between alveolar epithelial cells and the capillary network gets more attention. Fortunately, the lung-on-a-chip model could replicate this alveolar-capillary interaction by integrating tissue–tissue interfaces and may be crucial for the systemic understanding of COVID-19.

Organ-on-a-chip is a novel 3D cell culture tool based on the integration of the techniques of bioengineering and microfluidics disciplines (Shrestha et al., 2020). It always consists of upper and lower microchannels separated by a thin, flexible, extracellular matrix (ECM)-coated membrane, which is very suitable for mimicking the alveolar-capillary interface (Huh et al., 2010; Kızılkurtlu et al., 2018). For example, when human alveolar epithelial cells and pulmonary microvascular endothelial cells are cultured on the opposite sides of the membrane and grown to confluence, the upper channel is introduced into the air to create an air-liquid interface. In addition, a computer-controlled vacuum in these chambers can be used to produce cyclic strain ranging from 5% to 15% to match normal levels of strain to mimic physiopathological breathing movements (Birukov et al., 2003). Therefore, organ-on-a-chip can manipulate not only biochemical factors (such as cytokines, oxygen, and nutrients) but also dynamic physical factors (such as shear stress and cyclic strain), both of which are critical in understanding lung organ-level functions and permit researchers to mimic disease pathogenesis of COVID-19 (Huh et al., 2010; Benam et al., 2016; Novak et al., 2021; Wang et al., 2022).

**FIGURE 3:**
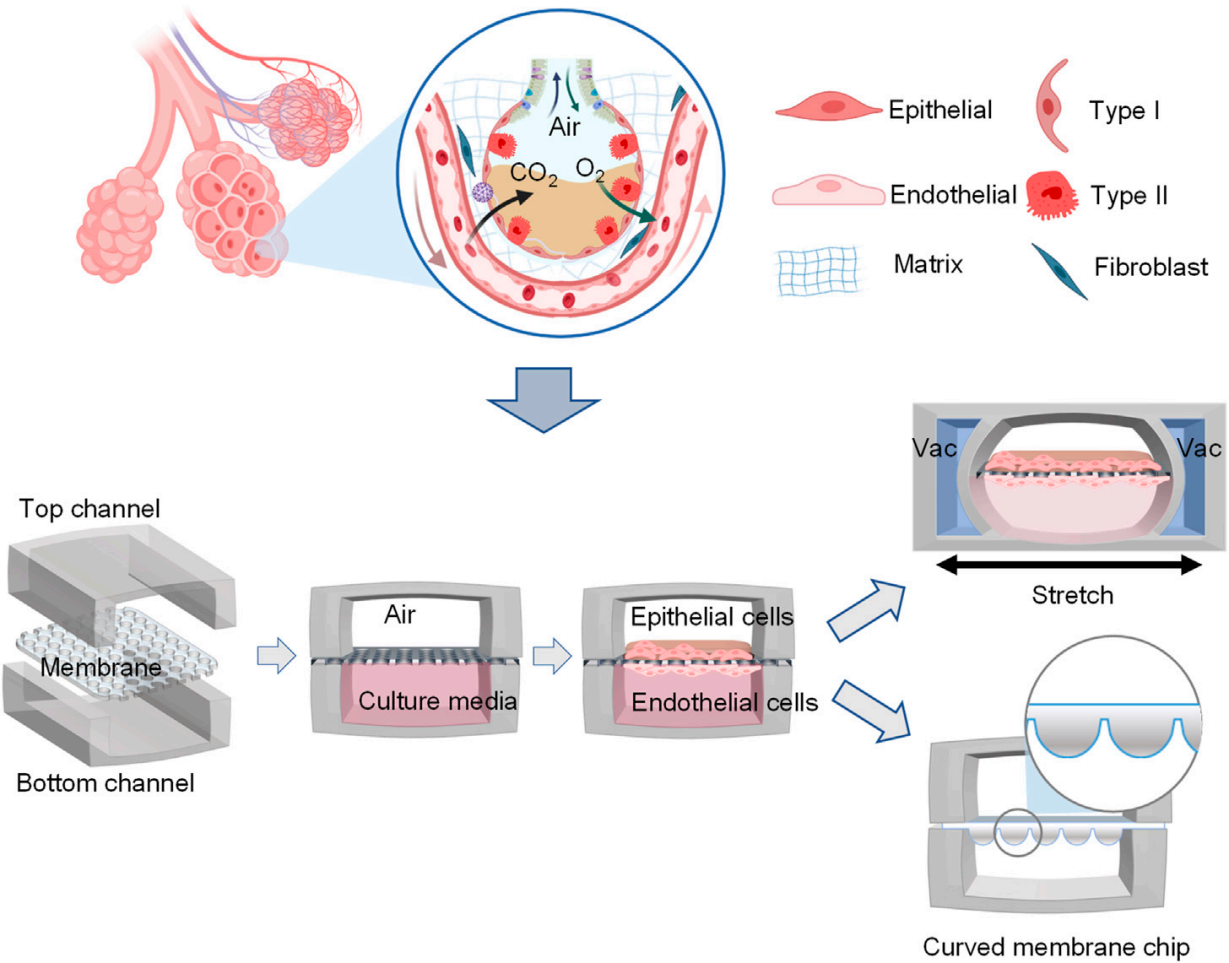
Schematic representation of functional lung-on-a-chip based on epithelium-endothelium interface physiology characteristic. During normal inspiration, pressure pulls air into the lungs, resulting in stretching of the alveolar epithelium. For mimicking this phenomenon, pressure-driven stretching by incorporating two larger, lateral microchambers into the device design (Huh et al., 2010). Also, microscale curved surfaces affect the spatiotemporal organization and behaviors of cells. The membrane was given the shape of hexagonally arrayed hemispherical microwells into the device design (Baptista et al., 2022).

In addition to dynamic mechanical distortion of the alveolar-capillary interface, there is increasing evidence that the microscale curved surfaces affect the spatiotemporal organization and behaviors of cells (Callens et al., 2020; Jin et al., 2021). To recreate the mainly spherical geometry of the cells’ native microenvironment, Baptista et al. (2022) made the membranes the shape of hexagonally arrayed hemispherical microwells by using a combination of 3D microfilm forming and ion track technology. Each microwell has a little bit more than 200 μm maximum inner diameter and an average maximum depth of 100.6 ± 3.0 μm. With integration in microfluidic chips, the microcurved membranes were seeded with Calu-3 lung epithelial cell line and human lung microvascular endothelial cells, respectively. Also, Huang et al. (2021) successfully designed an alveolar lung-on-a-chip platform with the alveoli-like 3D gelatin methacryloyl (GelMA) inverse opal structure. Significantly, this device also provided an air-liquid interface and cyclic strain, which was better able to maintain the functions of human alveolar epithelial cells. A list of characteristics and applications of current lung-on-a-chip devices is given in Table 3. A visual representation of these classifications is found in Figure 3.

**TABLE 3:** List for characteristics and application of current lung-on-a-chip devices.

Devices	Structures and seeding cells	Fabrication materials/Techniques	Characteristics	Aplication	Refs
Lung-on-a-chip	Two microchannels; the upper: human alveolar epithelial cells and the lower: microvascular endothelial cells	PDMS/soft lithography	Cyclic stretching: computer-controlled two larger, lateral vacuum microchambers to produce cyclic stretching (5 ∼ 15%)	Replicates dynamic mechanical distortion of the alveolar-capillary interface caused by breathing movements	Huh et al. (2010)
Lung-on-a-chip	Two microchannels; the upper: Calu-3 lung epithelial cell and the lower: microvascular endothelial cells	PC film/three-dimensional (3D) microfilm forming and ion track technology	The membranes with the shape of hexagonally arrayed hemispherical microwells (inner diameter: 200 μm, depth: 100.6 ± 3.0 μm)	Set the stage for other (micro) anatomically inspired membrane-based lung-on-a-chip models	Baptista et al. (2022)
Alveolar lung-on-a-chip	A three-dimensional porous hydrogel made of gelatin methacryloyl with an inverse opal structure; human alveolar epithelial cells	7% (wt/wt) GelMA/assemble alginate microbeads into a cubic close-packed lattice, infiltrate GelMA, remove alginate microbeads	Possess both the sac-like pores and the interconnecting windows between the sacs, in addition to a stiffness close to the native human lung	Investigates the effects of cigarette smoke and SARS-CoV-2 pseudoviral infection	Huang et al. (2021)
Airway-on-a-chip	Two microchannels; the upper: airway epithelial cells and the lower: airway smooth muscle cells	PMMA/Micromilling	Suspended hydrogels (type I collagen and Matrigel) as a middle layer to allow disassembly for downstream analyses	Significantly advance the understanding of smooth muscle cells–epithelial cells–matrix interactions	Humayun et al. (2018)
Airways-on-Chip	Three individual chips; nasal: RPMI-2650 cells, bronchial: Calu-3 cells, and acinar airways: hAELVi cell	PDMS/microfabrication and 3D printing	Mimics key elements of the respiratory system spanning (i) nasal passages, (ii) the mid-bronchial airway region and (iii) the deep acinar region, distinct with alveolated airways	Serve as a preclinical *in vitro* benchmark underlining regional lung crosstalk for viral infection pathways	Nof et al. (2022)

Abbreviations: PDMS, polydimethylsiloxane; PC, polycarbonate; GelMA, gelatin methacryloyl; SARS-CoV-2, severe acute respiratory syndrome coronavirus 2; PMMA, polymethylmethacrylate.

Multiple clinical trials have found that microvascular thrombotic and inflammatory processes may be crucial in exacerbating ARDS and increasing lung damage (Ackermann et al., 2020; Jung et al., 2020; Sardu et al., 2020). The vascular damage, and whether it is a direct consequence of endothelial infection or an indirect consequence of immune cells-mediated cytokine storm remain unclear. By using a vascularized lung-on-a-chip model, Thacker et al. (2021) found that infection of alveolar epithelial cells leads to the limited apical release of virions, and viral RNA and proteins are rapidly detected in underlying endothelial cells, which are themselves refractory to apical infection in monocultures. In addition, endothelial cells infected by SARS-CoV-2 lose expression of tight junction markers and adopt a pro-coagulatory phenotype. These results indicate that the dynamics of vascular damage are a direct consequence of endothelial infection independently of a cytokine storm.

To accurately resemble human-relevant responses to viral infection, Zhang M. et al. (2020) also created an alveoli-on-a-chip consisting of the upper human alveolar epithelium channel, lower microvascular endothelium, and circulating immune cells channel. They found a higher susceptibility to SARS-CoV-2 infection in the epithelium than in endothelium upon SARS-CoV-2 infection. Furthermore, they used RNA-seq analysis of immune responses to SARS-CoV-2 infection in this alveolus chip, and the results suggested the crucial role of immune cells involved in alveolar barrier injury and exacerbated inflammation. These results might explain the pathogenesis of the lung microvascular thrombosis and endotheliitis that existed in severe cases of SARS-CoV-2 infection.

With the advances in microfabrication technology, microfluidics, and tissue engineering (Jin et al., 2021), new approaches to the development of lung-on-a-chip models enable the production of more robust and high-throughput human *in vitro* respiratory tract models. The organoplate is an organ-on-a-chip platform comprising 96 microchambers that can be used for 3D cell culture (Trietsch et al., 2013). These microchambers are incorporated into a standard 384-well microtiter plate that is pipette-operatable and fully compatible with industrial readout and liquid handling equipment. Each microchamber consists of adjacent microchannels separated by phase-guides and four wells are linked together by microfluidic channels (Junaid and Hankemeier, 2021). van Duinen et al. (2017) used such organoplate to assess the vascular barrier function of 96 perfusable blood vessels which have a size-selective permeability with data from *in vivo* studies, and found that cytokines such VEGF and TNFα have dose-dependent effects on the vascular permeability.

In short, these studies show that lung-on-a-chip contributes to the exploration of the intricate cross-talk between vascular networks and alveolar epithelial cells.

### 3.5 3D bioprinting to mimic pulmonary interstitium

Although organoid and lung-on-a-chip models are suitable for mimicking the 3D structure and function of alveoli and airway as well as the interaction of alveoli and capillary networks, they cannot mimic the heterogenic pulmonary interstitium structures with complex components (consisting of collagen, elastin, fibronectin, glycoproteins, proteoglycans, 60 different kinds of cells) and certain arrangement (airways tree, alveoli network, and the vascular tree), which play an important role in controlling and guiding cellular behaviors to ultimately define tissue architecture (Suki et al., 2011; Barreiro Carpio et al., 2021; Novak et al., 2021).

3D bioprinting, an emerging novel 3D cell culture tool, deposits layer-by-layer cells and biomaterials in an organized and automatized manner (Zhang et al., 2017). Due to the capability of delivering cells and biomaterials with precise control over spatial distributions, it is possible to rapidly recreate engineered constructs with accurate architecture and composition of targeting tissues such as lungs, which are suitable for drug testing and virus infection (Malda et al., 2013; Murphy and Atala, 2014; Matai et al., 2020).

**FIGURE 4:**
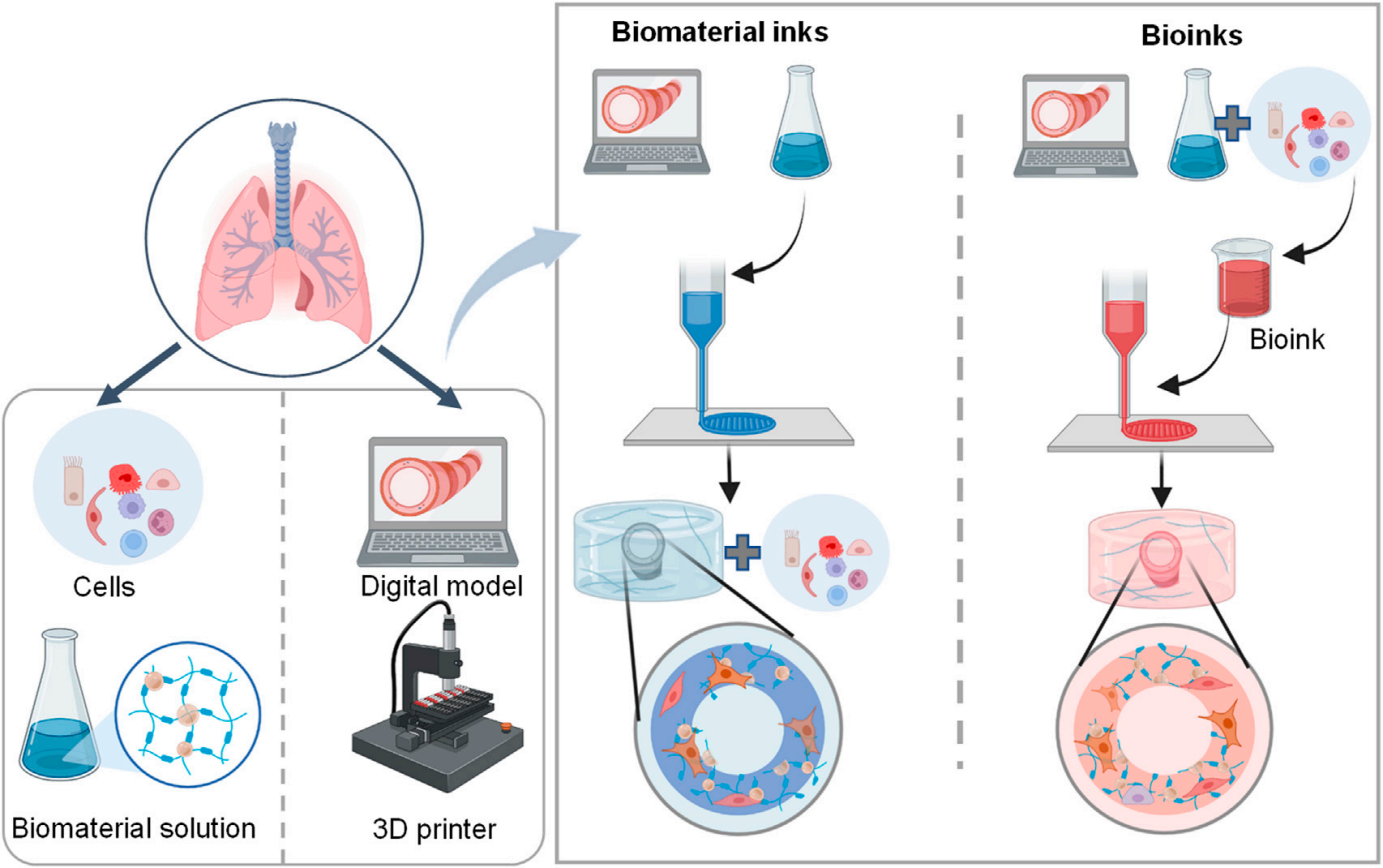
Schematic representation of the 3D lung bioprinting process. The general process of 3D bioprinting contains three steps: acquisition of a 3D CAD model, automated deposition of biomaterials (referred to biomaterial inks) or the mixture of cells and biomaterials (referred to bioinks), and maturation of cell-laden constructs to mimic desired tissue constructs (Barreiro Carpio et al., 2021).

The general process of 3D bioprinting contains three steps: acquisition of a 3D computer-aided design (CAD) model, automated deposition of biomaterials (referred to biomaterial inks) or the mixture of cells and biomaterials (referred to bioinks), and maturation of cell-laden constructs to reinforce the development of desired tissue constructs (Figure 4) (Mironov et al., 2003; Mironov et al., 2008; Zhang et al., 2017; Barreiro Carpio et al., 2021). There are different 3D printing strategies including extrusion-based bioprinting, inkjet/drop-on-demand, laser-assisted, stereolithography, and electronspinning-based bioprinting (Table 4). The most commonly used bioprinting is the extrusion-based technique, due to its ease of handling and low cost. The success of 3D bioprinting chiefly depends on the ability to formulate complex, cell-laden 3D structures. To date, there is no single bioprinting technique that enables the production of all scales and complexities of synthetic tissues and organs (Matai et al., 2020).

**TABLE 4:** List of some 3D bioprinting examples of different printing strategies.

Printing strategy	Cells/Bioink	Results	Application	Refs
Inkjet bioprinting	A549, EA.hy926 cells/Matrigel	Print the biofabrication of the human air-blood tissue barrier analogue composed of an endothelial cell, basement membrane and epithelial cell layer	An advanced 3D lung model for high-throughput screening for safety assessment and drug efficacy testing	Horvath et al. (2015)
Drop-on-demand	Human lung epithelial cells, human endothelial cells, and human lung fibroblasts/2.5% PVP	Cultured over a period of 14 days with high survivability rates	Fabricate 3D *in-vitro* alveolar lung models in an automated manner with high repeatability and reliability for studying respiratory diseases caused by infectious pathogens	Ng et al. (2021)
Two-photon stereolithography	phLFs/protein-based resins, such as bovine serum albumin or gelatin methacryloyl	Print up to mm-sized high-precision 3D cell scaffolds at micrometer with varying Young’s moduli ranging from 7–300 kPa	Allow for a systematic investigation ofsingle-cell and tissue dynamics in response to defined mechanical and bio-molecular cues	Erben et al. (2020)
Electrohydrodynamic printing	Mouse embryonic fibroblast cells and human non-small cell lung cancer cells/PCL/gliadin inks	The microstructure and the surface nanotopography of the printed scaffolds could be precisely controlled and turned	Provide the potential of cancer cell-seeded scaffolds as 3D *in vitro* tumor models for cancer research and drug screening	Jing et al. (2021)
Laser-assisted bioprinting	AR42J-B-13 rat acinar cell line/GELMA	Generate 3D pancreatic cell spheroid arrays	Provide a platform for the study of the internal and external factors that contribute to the formation of precursor PDAC lesions and to cancer progression	Hakobyan et al. (2020)

Abbreviations: A549, human alveolar epithelial type II cell line; EA.hy926, endothelial cells; PVP, polyvinylpyrrolidone; phLFs, primary human lung fibroblasts; PCL, poly-ε-caprolactone; GELMA, methacrylated gelatin; PDAC, pancreatic ductal adenocarcinoma.

To get high mm-sized high-precision scaffolds, Erben and co-workers (Erben et al., 2020) adopted two-photon stereolithography to print 3D cell scaffolds with varying Young’s moduli ranging from 7–300 kPa. The dynamics of colonizing primary human lung fibroblast cells are observed by modifying scaffold geometry. Also, Jing et al. (2021) successfully fabricated fibrous scaffolds *via* electrohydrodynamic printing. Based on drop-on-demand inkjet-printing, high-resolution deposition of alveolar cells enables to fabricate a three-layered alveolar barrier model with an unprecedented thickness of ≈10 μm, which better recapitulates the structure, morphologies, and functions of the lung tissue and could reproduce practical tissue-level responses to influenza infection (Kang et al., 2021).

Nowadays, more than 100 types of biomaterials are currently being used as bioinks for 3D bioprinting. The most important features of bioinks are bioactivity, biocompatibility, biodegradability, and mechanical properties which provide mechanical strength, physical stability, and biological features (Petersen et al., 2010; O’Brien, 2011; Nichols et al., 2014). Therefore, these bioinks are composed of many types of biocompatible materials from either natural or synthetic materials. Hydrogels such as collagen and GelMA are the most common scaffold to encapsulate cells since they are akin to the native ECM to enable cell differentiation and proper function (Zhao Z. et al., 2020). Studies suggested that some patients with severe COVID-19 develop fibrotic lung disease, an interstitial lung disease characterized by the excessive deposition of ECM proteins in the lung (Bharat et al., 2020; Tale et al., 2020). When the virus binds to the ECM components, it may significantly affect cellular responses to assist its infection in the host (de Melo et al., 2021). A good selection of bioink is of great significance for cell growth and the establishment of disease models. For example, Falcones et al. (2021) successfully designed a new bioink with tunable stiffness based on decellularized porcine lung ECM hydrogels for 3D culturing of lung-resident MSCs. This bioink doesn’t need additional chemical or physical crosslinking and improves preconditioning MSCs for therapeutic purposes.

These elements provide a basis for mimicking the heterogenic pulmonary interstitium structures. Horvath et al. (2015) reported the 3D bioprinting of an alveolar barrier by using a micro-extrusion bioprinter. The biofabricated structure was morphologically similar to the native tissue, being highly organized in a thin layer. On the other hand, cells manually mixed to Matrigel formed multi-layered clusters with tick ECM between the epithelial and endothelial cells, which can affect the permeability of biomolecules. Using 3D bioprinting *via* photopolymerizable hydrogels, Grigoryan et al. (2019) successfully designed the most complex alveolar models, which contain elaborate entangled vascular networks from space-filling mathematical topologies and can be used to explore the oxygenation and flow of human red blood cells during tidal ventilation and distension of a proximate airway.

To study the mechanisms of SARS-CoV-2 infection in the lung, the alveolar tissue can be mimicked using a 3D bioprinting model with high repeatability and reliability. Ng et al. (2021) reported the fabrication of human triple-layered alveolar lung models using the drop-on-demand 3D bioprinting. These 3D bioprinted human triple-layered alveolar lung models consisted of human lung epithelial cells, human endothelial cells, and human lung fibroblasts and showed high survivability rates over a long-term period of at least 14 days. This would help to address the demand for highly repeatable and scalable fabrication of 3D *in-vitro* alveolar lung models for studying global respiratory diseases caused by infectious pathogens. Also, in order to clarify virus detection and characterization, Koban et al. (2020) designed easy-to-handle 3D bioprinting platforms based on Wellbrick matrix containing gelatin and collagen additives. This will provide a promising way for the characterization of virus infections due to sensitive monitoring virus-host interactions and replication of different viruses under physiologically relevant conditions.

## 4 Application of 3D cell culture models in study of COVID-19 treatments

3D cell culture models can provide robust support to simulate SARS-CoV-2 infection, replication, and related immuno-inflammatory responses in humans, which is crucial to understanding the virus’s biology and developing antiviral drugs. For example, in addition to investigating viral effects on human pulmonary tissue, lung organoids are very helpful for high-throughput assays to screen therapeutic drugs, such as RNA-seq analyses, proteomics, phosphoproteomics, transcriptomics, and molecular docking analysis (Elbadawi and Efferth, 2020; Hekman et al., 2020). Moreover, organoids derived from patient lung tissue may lead to a promising resource of potentially effective drugs for treating and managing the disease (Xu et al., 2021). This process from the organoid establishment to drug testing is concise so that the organoid models will be a valuable platform for screening patient-specific drugs (Zhou et al., 2018; Sachs et al., 2019). Apart from organoid, a spheroid culture system for human alveolus was established and this platform has been used for accurate pre-clinical testing of candidate drugs for the treatment of COVID-19 (Ebisudani et al., 2021).

ACE2 is the canonical entry receptor used by SARS-CoV-2 yet is expressed in only a small fraction of airway epithelial cells, predominantly AT2 (Zhao B. et al., 2020). For example, by using hESC-derived cardiac and lung organoids, 1,443 FDA-approved drugs were used to search for modulators of ACE2 levels and identified that inhibitors of 5 alpha reductases, which inhibit androgen signaling, can reduce ACE2 levels in the target cells and thereby decrease SARS-CoV-2 infectivity (Samuel et al., 2020).

However, ACE2 alone cannot explain the multi-organ tropism of SARS-CoV-2, and some analyses of patients with COVID-19 revealed many virus-positive cells without ACE2 expression (Chua et al., 2020; Hikmet et al., 2020; Ren et al., 2021). This evidence indicates other receptors involved in SARS-CoV-2 host interactions. By using lung organoids that provide more physiological conditions, KREMEN1/ASGR1 and Tetraspanin 8 (TSPAN8) were identified as alternative functional receptors of SARS-CoV-2 (Hysenaj et al., 2021; Gu et al., 2022). Adult human stem cell-derived alveolosphere has been developed to provide long-term expansion and differentiation of human alveolar type 2 cells/pneumocytes, which express ACE2 and TMPRSS2. These organoids respond to SARS-CoV-2 infection with upregulation of IFN and downregulation of surfactant, and low-dose IFN blocks SARS-CoV-2 replication, which mimics the features of COVID-19 lungs (Katsura et al., 2020). These newly discovered host receptors play essential roles in ACE2-independent virus entry and potential therapeutic targets for COVID-19.

COVID-19 patients who are hospitalized frequently manifest lymphopenia, which suggests suppression of cellular immune responses (Zhang J. J. et al., 2020; Wang D. et al., 2020). 3D models mimicking the lung structure and function have great advantages to investigate airway immune responses to viral infection. For example, Nelli et al. (2016) developed an ALI culture system that closely mimics the natural airway epithelium to characterize the innate immune response of feline herpesvirus-1 (FHV-1). In addition, Purwada et al. (2015) described a B cell follicle organoid to control the rate of immune reaction through tunable design parameters. The next major development may be organoids of the thymus and lymph node because the thymus and bone marrow are the central immune organs for development, differentiation, and maturation of human immune cells.

COVID-19 patients in severe cases may also develop complications such as hypercoagulopathy, systemic endotheliitis, and even multi-organ failure, which are concomitant with a sustained release of inflammatory factors caused by an excessive inflammatory response. Such phenomenon of elevated levels of inflammatory factors has been detected in organoid models following SARS-CoV-2 infection (Mills et al., 2021; Pei et al., 2021). SARS-CoV-2 is also likely to enter vascular endothelial cells through infected airway epithelial cells, which may lead to endothelial dysfunction (Harrison et al., 2020). More recently, Hashimoto et al. (2022) used a lung-on-a-chip model consisting of two microchannels to mimic the interactions between epithelial, endothelial, and immune cells, and they demonstrated in this model that Claudin-5 is a key factor in disrupting vascular endothelial cadherin-mediated adherent junctions.

In clinical trials different strategies have been adopted to deal with an excessive inflammatory response. They either boost the early type I or III interferon responses in order to help patient get rid of the virus before it causes hyper-inflammation, or suppress immune response by inhibiting specific pro-inflammatory pathways such as IL-6. However, it is still in question how to balance the immune status so that the viral replication can be effectively suppressed without cytokine storm to cause organ failure. Correct use of 3D models may help better understand this question and thus accelerate optimization of the immune therapeutic approach for SARS-CoV-2 infection.

Furthermore, 3D lung models serve as a powerful platform for drug screening and safety assays against SARS-CoV-2, targeting host cells factors (ACE2, TMPRSS2, ACAT and HIF1α) and the virus itself (Tiwari et al., 2021; Kastenhuber et al., 2022). By combining iPSCs-derived lung organoids and high-throughput screening techniques from United States Food and Drug Administration (FDA)-approved drugs, GW6471 has been identified to block SARS-CoV-2 infection (Duan et al., 2021) and imatinib, mycophenolic acid (MPA), and quinacrine dihydrochloride (QNHC) have been identified to block SARS-CoV-2 entry (Han et al., 2021). Combining iPSCs-derived lung organoids with differentiated AT2 cells and a connectivity mapping approach, atorvastatin was predicted to be the most promising candidate from 20,000 small compounds for blocking SARS-CoV-2 (Duarte et al., 2021).

Remdesivir has been recently recognized as a promising antiviral drug against many RNA viruses (e.g., SARS, MERS-CoV), including SARS-CoV-2 (Wang M. et al., 2020). By using an air-liquid interface model, remdesivir has been reported to decrease the colony-forming efficiency (CFE) of club cells, but promote the growth of club organoids (Wang J. et al., 2020). Also, by using an alveolar chip, remdesivir has been proven to inhibit viral replication and alleviate the disruption of the alveolar-capillary barrier (Zhang M. et al., 2020).

**FIGURE 5:**
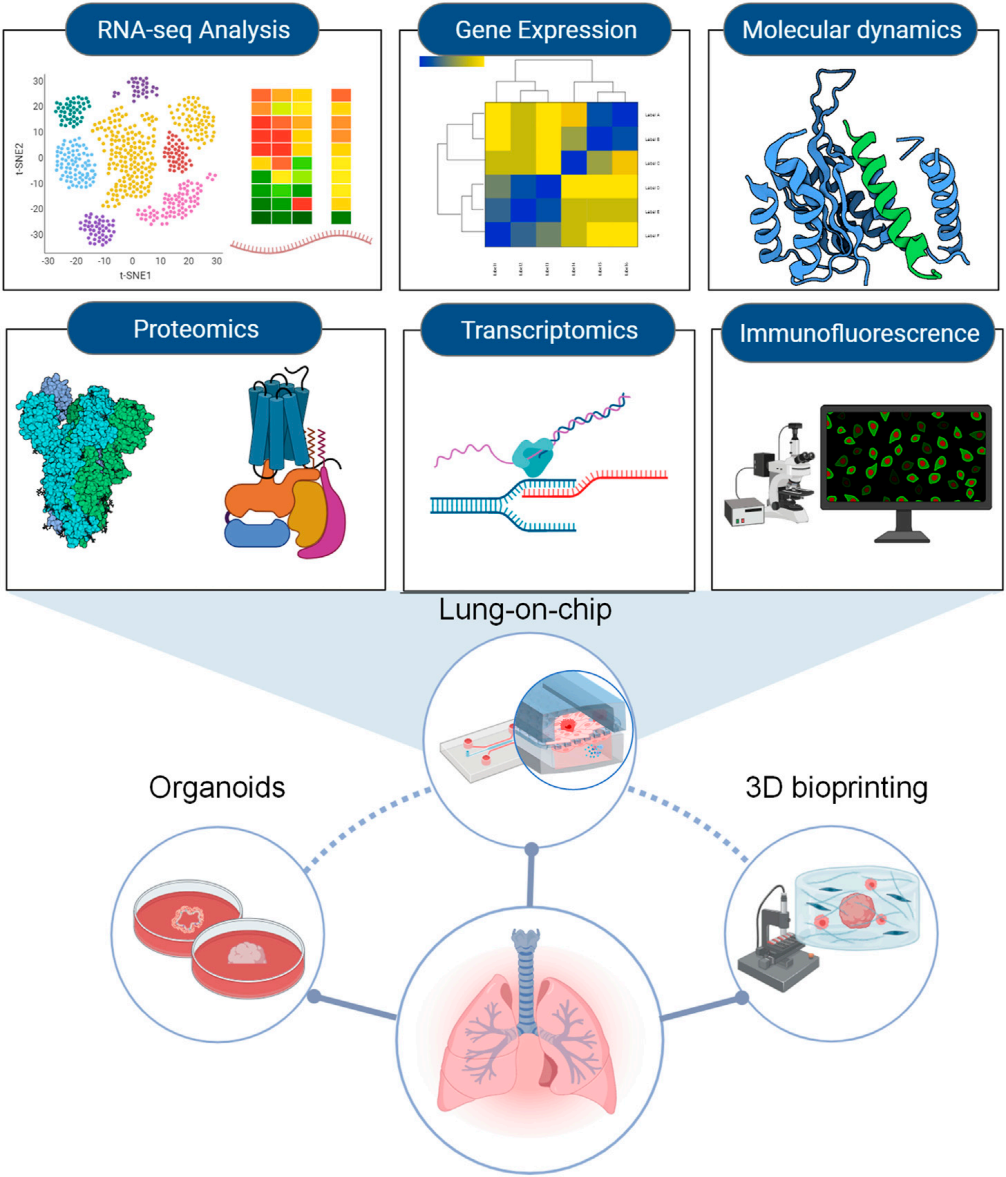
An overview of assays for screening agents to combat SARS-CoV-2 with 3D models.

A systematic example of 3D cell culture models with some fast-track approaches used in screening drugs against COVID-19 is listed in Figure 5. A summary of the aforementioned drugs suitable for COVID-19 treatment can be seen in Table 5.

**TABLE 5:** Drugs against SARS-CoV-2 discovered/tested by human lung organoids.

Names	Combined with screening/Sources of drugs	Objective targets	Mechanism	Concentration	Cells for construct organoids	Refs
Antiandrogenic drugs	A Selleckchem small molecule library	ACE2	Reduce ACE2 expression	1 μM, 2 μM or 5 μM	hESCs	Samuel et al. (2020)
25HC	A natural product	ACAT	Block membrane fusion	5 μM	iPSCs	Wang et al. (2020e)
GW6471	Agonists and antagonists and FDA-approved drugs	HIF1α	Inhibit the HIF1a-glycolysis axis	EC50 = 2.1 μM	hESCs	Duan et al. (2021)
Atorvastatin	A connectivity mapping approach in combination with chemoinformatic analyses		Bind to SARS-CoV-2’s main protease and RNA-dependent RNA polymerase	IC50 values of 31.65 μM	hESCs	Duarte et al. (2021)
K-874A	VHH-cDNA display	SARS-CoV-2 S1 proteins	Block the virus membrane from fusing with the host cell membrane	IC50 values of 5.74 ± 2.6 μg/ml	Normal lung tissues	Haga et al. (2021)
Ciclesonide, nelfinavir, and camostat	FDA-approved drugs, bioactives kinase inhibitors, and natural products				iPSCs	Ko et al. (2021)
Lead E24	Medicinal chemistry and rational drug design strategies	SARS-CoV-2 main protease	Bind SARS-CoV-2 M^pro^ and inhibit proteolytic activity	EC50 values of 844 nM	iPSCs	Huff et al. (2022)
DPP4 peptide; Ab15033-7		DPP4 receptor; Spike protein		DPP4 peptide (200 µg) or Ab15033-7 (200 ng/ml)	iPSCs	Spitalieri et al. (2022)

Abbreviations: SARS-CoV-2, Severe Acute Respiratory Syndrome-CoronaVirus-2; ACAT, activating acyl-CoA, cholesterol acyltransferase; DPP4, dipeptidyl peptidase-4; 25HC, 25-hydrocholesterol; BALF, bronchoalveolar lavage fluid; ACE2, angiotensinconverting enzyme 2; FDA, US food and drug administration; HIF1a, hypoxia-inducible factor 1 subunit alpha; iPSCs, induced pluripotent stem cells; AT2s, alveolar epithelial type-2 cells; ESCs, embryonic stem cells.

## 5 Future direction and perspectives

Since the complexity of SARS-CoV-2 infection and the pathogenesis of COVID-19 and the timeliness for high-throughput of drug screening for therapeutic treatment, the use of 3D cell culture toolsets provides increasing similarity to the *in vivo* pathophysiology and may give us valuable insights and more reliable tools for drug development and testing in the treatments of COVID-19 (Ravi et al., 2015).

Recent advances in organoids have enabled the modeling of SARS-CoV-2 infection using human airway epithelial and alveolar cells. These culture models, however, lack airway smooth muscle cells (ASMCs). ASMCs are known to switch between contractile and proliferative phenotypes in response to various physical and chemical cues, which play essential roles in lung development and respiratory diseases (Kim et al., 2015). Therefore, organoids containing ASMCs may better recapitulate the airway structure and function *in vivo*, and if co-cultured with immune cells, could be used to reveal more detailed mechanisms of the COVID-19-associated immunology.

Appropriate culture media should also be developed for efficient generation and maintenance of lung organoids. Such media should contain not only basal medium, but also a variety of growth factors and small-molecules. For example, TGF-β has an anti-inflammatory effect on cell response induced by influenza H1N1 virus (BustosRivera-Bahena et al., 2021). TGF-β also inhibits cell proliferation and promotes apoptosis and differentiation. So far, effect of TGF-β in the culture medium on efficiency of organoid formation has been evaluated (Shi and Massague, 2003), and evaluation may be required of other signaling pathways such as Wnt/β regarding their effects on organoid culture medium.

Lung-on-a-chip is always restricted to mimic the alveolar-capillary barrier and cannot be used to resemble the airway tubular structure. In addition, lung-on-a-chip is typically limited by the number of cells as compared to that *in vivo*, which may lead to altered cellular function such as the metabolic rate. Such issues need to be addressed in the development of next-generation lung-on-a-chip systems.

3D bioprinting airway or alveoli is always expensive, which leads that it is almost impossible to set up high-throughput approaches for drug screening using these 3D cell culture models. In addition, despite the extensive use of 3D cell culture models in basic research, their translational biomedical application is restricted since the lack of robust, reproducible, and scalable methods of production in compliance with current pharmaceutical standards. The reproducibility, accuracy, and scalability of the methodologies proposed still need to be improved (Vives and Batlle-Morera, 2020). Therefore, easy-to-use, rapid, and low-cost strategies in the fabrication of 3D cell culture models with more complex cytoarchitecture and a more physiological microenvironment still need to develop.

Despite of their potential, 3D models still entail considerable technical problems that may compromise their application, which include but are not limited to sample collection, high-quality imaging, and cost of substrate materials such as Matrigel. For example, it is technically challenging to directly collect samples from Matrigel-based 3D models such as organoids or 3D bioprinting because direct sample collection would disrupt the integrity of Matrigel and thus the structure of the 3D model, leading to inaccurate results. It is also difficult to obtain high-quality imaging of these 3D cultures by using traditional imaging techniques such as paraffin embedding and tissue slicing (Rios and Clevers, 2018). Although non-invasive microscopy methods such as multi-photon and light-sheet microscopy can be used to visualize cellular details of 3D cultures, the thick sample can cause light scattering because of mismatched refractive indices. To reduce scattering within the sample, various optical clearing methods have been developed. For example, Dekkers et al. (2019) successfully designed a simple optical clearing method utilizing a homemade fructose-glycerol clearing agent to improve the light penetration through fixed organoids, whereas Boothe et al. (2017) used another agent named Iodixanol for the same purpose.

3D models commonly use Matrigel (Corning) or Cultrex BME (Trevigen) as substrate materials. These materials are too expensive to be used in large-scale studies such as high-throughput screening, regenerative medicine and diagnostics (Curvello et al., 2020). This high cost may be reduced by replacing Matrigel/Cultrex BME with an alternative in the 3D models. For example, gels made of decellularized porcine small intestine mucosa/submucosa were used to generate human gastric organoids (Giobbe et al., 2019). Recently, a novel engineered plant-based nanocellulose hydrogel was developed to provide the required microenvironment for small intestinal organoid growth and budding (Curvello et al., 2020).

Materials properties and fluid dynamics are also important issues to consider in developing 3D cultures. For example, Matrigel needs to be mixed with agarose to achieve proper mechanical properties for long time maintenance of a tubular organoid structure (Güney et al., 2021). In order to provide the low shear environment suitable for iPSCs differentiation, organoids can be cultured in spinner flasks, which can be further combined with microfluidic design to achieve both chemical and mechanical stimulation in tunable fashions.

Last but not least, each of the 3D models has its own advantages and disadvantages, and it may be feasible to combine some of these models as a novel platform with enhanced functionality. For example, although lung organoids always automatedly generate target tissue structure and function, they lack typical epithelium-endothelium tissue interfaces *in vivo* and are time-consuming to fabricate. On the other hand, the advantage of 3D bioprinting is the possibility to precisely define the composition and arrangement of a culture which can be realized rapidly. Combining the organoid and 3D bioprinting techniques may establish a new cell culture model to reflect the near-physiological cross-talk among airway and vascular vessels after SARS-CoV-2 infection. Advances in bioink preparation including the incorporation of bioactive matrices, and induced pluripotent stem cells, and suitable induction factors may open up broad applications for 3D cell culture models in disease diagnostics, and regenerative medicine.

## 6 Conclusion

The COVID-19 pandemic has resulted in global health and economic burden, but the underlying pathogenesis and therapeutic treatments remain to be further explored. Novel 3D cell culture models representing the lung structure and function in a dish that is in good agreement with reports from animal models and clinical disease are undoubtedly a valuable toolset for these studies, which may provide a promising alternative for animal models and 2D cell culture models. This review of the strategies to fabricate useful 3D pulmonary cell culture models including organoids, lab-on-a-chip, and bioprinting, although by no means comprehensive, indeed provide new insights for understanding how SARS-CoV-2 infects human lung cells, which is essential for elaborating the virus-induced human responses and helping further development of novel therapeutics and prophylactics for COVID-19.

## Glossary

2D: Two-dimensional
3D: Three-dimensional
ACE2: Angiotensin-converting enzyme 2
AFE: Anterior foregut endoderm
ALI: Air liquid interface
APCs: Antigen presenting cells
ARDS: Acute respiratory distress syndrome
ASMCs: Airway smooth muscle cells
AT2: Alveolar epithelial type 2
CAD: Computer-aided design
CFE: Colony-forming efficiency
COVID-19: Coronavirus disease 2019
DE: Definitive endoderm
ECM: Extracellular matrix
ESCs: Embryonic stem cells
FDA: Food and Drug Administration
FHV-1: Feline herpesvirus-1
GelMA: Gelatin methacryloyl
IL: Interleukin
iPSCs: Induced pluripotent stem cells
LPs: Lung progenitors
MDA5: Melanoma differentiation-associated gene 5
MBCS: Multibasic cleavage site
MPA: Mycophenolic acid
NHPs: Non-human primates
NRP1: Neuropilin-1
ORFs: Open reading frames
PC: Pyruvate carboxylase
PD: Peptidase domain
PM2.5: Fine particulate matter
QNHC: Quinacrine dihydrochloride
RBD: Receptor-binding domain
SARS-CoV-2: Severe acute respiratory syndrome coronavirus-2
TLR3: Toll-like receptor 3
TLR7: Toll-like receptor 7
TMPRSS2: Transmembrane protease serine 2
TNF-a: Tumor necrosis factor alpha
TSPAN8: Tetraspanin 8
